# Does ^18^F-FDG Positron Emission Tomography-Computed Tomography Have a Role in Initial Staging of Hepatocellular Carcinoma?

**DOI:** 10.1371/journal.pone.0105679

**Published:** 2014-08-25

**Authors:** Yuri Cho, Dong Hyeon Lee, Yun Bin Lee, Minjong Lee, Jeong-ju Yoo, Won-mook Choi, Young Youn Cho, Jin Chul Paeng, Keon Wook Kang, June-Key Chung, Su Jong Yu, Jeong-Hoon Lee, Jung-Hwan Yoon, Hyo-Suk Lee, Yoon Jun Kim

**Affiliations:** 1 Department of Internal Medicine and Liver Research Institute, Seoul National University College of Medicine, Seoul, Republic of Korea; 2 Department of Nuclear Medicine, Seoul National University Hospital, Seoul, Republic of Korea; University of Pavia, Italy

## Abstract

**Background and Aim:**

The utility of fluorine-18 fluorodeoxyglucose (^18^F-FDG) positron emission tomography-computed tomography (PET/CT) in initial staging of hepatocellular carcinoma (HCC) has yet to be fully explored. We assessed the usefulness of ^18^F-FDG PET/CT in initial staging of HCC.

**Methods:**

A total of 457 consecutive patients initially diagnosed with HCC at Seoul National University Hospital between 2006 and 2012 were evaluated retrospectively to assess the impact of ^18^F-FDG PET/CT on staging and compliancy with Milan criteria, relative to dynamic CT of liver and chest x-ray.

**Results:**

Seven among the 457 patients studied showed a shift in Barcelona Clinic Liver Cancer [BCLC] stage (A→C: 6 patients; B→C: 1 patient) and 5 patients who had originally met Milan criteria no longer qualified. ^18^F-FDG PET/CT had value in initial staging of early (stage A) or intermediate (stage B) HCC, as determined by dynamic CT of liver and BCLC or AJCC classifications, whereas BCLC stage 0 and stage C tumors were unchanged (*P*<0.001). ^18^F-FDG PET/CT disclosed additional metastases in patients with American Joint Committee on Cancer [AJCC] T2 (2.7%), T3a (5.3%), and T3b (4.8%) classifications.

**Conclusions:**

In initial staging of HCC, ^18^F-FDG PET/CT provided additional information, impacting the patients with BCLC (stages A and B) and AJCC (T2 and T3) classifications. Its use might be thus appropriate for these patient subsets, especially if hepatic resection or liver transplantation is planned.

## Introduction

Hepatocellular carcinoma (HCC) is among the most prevalent cancers worldwide, ranging from 30 per 100,000 population in Southeast Asia to 2 per 100,000 in northern Europe and the United States [1]–[3], and extrahepatic involvement is not unusual [4], [5]. However, newer treatment modalities are currently emerging for those afflicted with HCC [6]–[8]. When tumor is confined to the liver, aggressive locoregional therapy or liver transplantation (LT) are options, provided the Milan criteria are met. However, when the patients have metastatic HCC, locoregional treatment including LT has limited efficacy [9]. Sorafenib is presently the only treatment with proven survival benefit (relative to best supportive care) in instances of extrahepatic spread [10]. Extrahepatic HCC is thus regarded as systemic disease, with a poor prognosis, and constitutes a contraindication to LT. Hence, the need for accurate staging of HCC, so that treatment may be optimized.

Beyond the liver, HCC frequently involves lungs, abdominal lymph nodes (LN), and bones [11]. Various imaging modalities, including ultrasonography, computed tomography (CT), magnetic resonance imaging (MRI), positron emission tomography (PET), bone scan, chest x-ray, and angiography, are used in the staging of HCC [12], [13]. Although liver dynamic CT and MRI are particularly useful in diagnosing intrahepatic HCC or intra-abdominal lymph node metastases, detection of distant metastases (ie, in brain or bone) can be more problematic [14]. Current guidelines for HCC recommend abdominal dynamic CT or MRI, chest CT, and bone scintigraphy for pre-operative staging [15]. However, the role of PET-based imaging in preoperative testing has yet to be fully explored.

PET using fluorine-18 fluorodeoxyglucose (^18^F-FDG) is a useful tool for detecting distant metastases in other malignancies [16]. Although the role of ^18^F-FDG PET in HCC has been investigated to a degree [17], [18], the small populations recruited may not be representative. In this study, we assessed the utility of ^18^F-FDG PET/CT in the initial work-up (dynamic CT of liver and chest x-ray) of HCC by determining its impact on staging (Barcelona Clinic Liver Cancer [BCLC] and American Joint Committee on Cancer [AJCC] TNM [7^th^ edition] conventions) and compliancy with Milan criteria. We also examined any subsequent ramifications for treatment.

## Methods

The study protocol was reviewed and approved by the Institutional Review Board of Seoul National University Hospital (IRB No. 1309-040-519). This study is a retrospective analysis. Therefore, we could not obtain the written informed consents. The data were analyzed anonymously.

### Patients

A total of 3327 consecutive patients initially diagnosed with HCC at Seoul National University Hospital between April, 2006 and December, 2012 were reviewed retrospectively. The diagnosis was established through histology or was based on the non-invasive diagnostic criteria of the American Association for the Study of Liver Diseases (AASLD) [19]. Among all the consecutive patients, 457 patients who underwent ^18^F-FDG PET/CT at the time of HCC diagnosis were included in this study. ^18^F-FDG PET/CT was performed for the patients who had been considered to be treated with surgical resection, locoregional therapies (ie, percutaneous ethanol injection, radiofrequency ablation or LT), and for some patients with aggressive rapid-growing HCC who had been considered to be treated with transarterial chemoembolization (TACE).

### Radiologic and scintigraphic studies

All patients underwent triple-phase dynamic CT of liver or contrast-enhanced MRI of the abdomen (to evaluate intrahepatic HCC and extrahepatic metastases), supplemented by chest x-ray (to detect lung metastases). None of these patients had received prior treatment for HCC.

^18^F-FDG PET/CT (GE Advance, Milwaukee, WI, USA) was performed to detect occult extrahepatic metastasis. All patients fasted at least 6 hours beforehand. Approximately 370 MBq of ^18^F-FDG was injected intravenously, and 50–60 min later, an emission scan (5 min/bed position) in 2-dimensional mode was performed from the neck to the knees. This was followed by a transmission scan (3 min/bed position), done via rotating 68-Ge source. The images were then reconstructed, utilizing the attenuation-corrected ordered subset expectation maximization algorithm (16 subsets, 2 iterations). ^18^F-FDG PET/CT images were subsequently compared with corresponding CT or MRI images in terms of tumor localization. Two specialists in nuclear medicine evaluated coronal, sagittal, and axial images qualitatively to verify that ^18^F-FDG FDG tumor uptake surpassed that of surrounding noncancerous tissue.

Extrahepatic metastases were confirmed initially by radiologic means and were monitored at least 3 months after ^18^F-FDG PET/CT by follow-up imaging, including chest CT, abdominal CT/MRI, and whole-body bone scan or MRI of bone.

### Impact of ^18^F-FDG PET/CT on HCC stage

The BCLC and AJCC TNM classifications were assigned before and after ^18^F-FDG PET/CT, respectively. Lesions on ^18^F-FDG PET/CT that had gone undetected by dynamic CT of liver and chest x-ray were analyzed relative to BCLC stage, AJCC T classification, and compliancy with Milan criteria. To determine the impact of ^18^F-FDG PET/CT on treatment decisions, we examined shifts in BCLC stage or compliancy with Milan criteria that would alter patient management.

Various parameters assessed included gender; age; etiology of liver disease; Child-Pugh classification; alpha-fetoprotein (AFP) levels; protein induced by vitamin K antagonist II (PIVKA-II) levels; number (single or multiple), shape (nodular or infiltrative), and size (maximum diameter) of primary tumor(s); and presence of portal vein thrombosis.

### Statistical Analysis

All analyses were conducted using standard statistical software (SPSS 12.0 for Windows, SPSS Inc, Chicago, IL, USA). Baseline patient clinical parameters were expressed as medians (range) and frequencies. Chi-square and Fisher's exact tests were used for comparing clinical parameters and tumor characteristics. A two-tailed *P*-value <0.05 was considered statistically significant.

## Results

### Baseline Characteristics

Baseline characteristics of the 457 patients are shown in Table 1. Median age was 53.8 years (range, 28–72 years), and 365 patients (79.9%) were male. A majority of the tumors were attributable to hepatitis B virus infection (386, 84.5%). In most patients, liver function and performance status were favorable (Child-Turcotte-Pugh [CTP] class A: 364, 78.3%; ECOG score of 0: 367, 80.3%). Of the 457 patients, 397 (86.9%) had clinical manifestations of liver cirrhosis (LC) including thrombocytopenia, splenomegaly, esophageal/gastric varices, or ascites. All of the patients (n = 25) with chronic hepatitis C infection showed clinical manifestations of LC. Among the patients with chronic hepatitis B infection, 84.5% (326 of 386 patients) showed clinical manifestations of LC at the time of HCC diagnosis.

**Table 1:** Baseline characteristics.

Variables	Total (n = 457)
Median age, year (range)	53.8 (28–72)
Male sex, n (%)	365 (79.9)
Etiology, n (%)	73 (51.4)
HBV/HCV/alcohol/others	386/25/16/30 (84.5/5.5/3.5/6.6)
CTP classification, n (%)	
A/B/C	364/68/25 (78.3/14.6/5.4)
ECOG (PST), n (%)	
0/1/2	367/84/6 (80.3/18.4/1.3)
Tumor number, n (%)	
Single/multiple	237/220 (51.9/48.1)
Median tumor size (cm) (range)	4.1 (0.8–18)
Tumor type, n (%)	
Nodular/infiltrative	427/30 (93.4/6.6)
Vascular tumor invasion, presence, n (%)	116 (25.4)
Portal vein/hepatic vein or IVC/both	91/10/15 (19.9/8.6/3.3)
Median alpha-fetoprotrein (ng/mL) (range)	128.7 (5–1708000)
Median PIVKA-II (mAU/mL) (range)	53.2 (6–48000)

Abbreviation: CTP, Child-Turcotte-Pugh classification; ECOG, Eastern Cooperative Oncology Group; HBV, hepatitis B virus; HCV, hepatitis C virus; IVC, inferior vena cava; PST, performance status; PIVKA-II, Prothrombin Induced by Vitamin K Antagonist-II.

Approximately 72% of the patients (329 of 457 patients) had been under regular surveillance for HCC performing liver ultrasonography or dynamic CT of liver and serum AFP every 6 months due to the high risk factors of HCC (ie, chronic hepatitis B infection, hepatitis C virus [HCV]-related LC, alcoholic LC).

At the time of diagnosis, 237 patients (51.9%) had isolated tumors, whereas in 220 (48.1%), HCC was multifocal. Median tumor size was 4.1 cm (range, 0.8–18 cm), with nodular configurations (93.4%) predominating, and 116 lesions (25.4%) displayed gross vascular invasion. Median serum AFP and PIVKA-II concentrations were 128.7 ng/mL (range, 5–1.7×10^6^ ng/mL) and 53.2 mAU/mL (range, 6–48000 mAU/mL), respectively (Table 1).

### Metastatic lesions on ^18^F-FDG PET/CT

Initial ^18^F-FDG PET/CT findings were abnormal in 219 (47.9%) of the 457 patients. Benign lesions, such as non-specific inflammation, bronchiectasis, pneumonia, pulmonary tuberculosis, and bulla, were observed in 174 patients (38.1%), with lung most frequently implicated (55, 12.0%). Other affected sites included LN, bone, gastrointestinal tract, pancreas, thyroid gland, prostate gland, adrenal gland, peritoneal cavity, parotid gland, and spleen (Table 2).

**Table 2:** Metastatic lesions observed on ^18^F-FDG PET/CT.

Findings on PET/CT	Number (n = 457)	%
**Abnormal lesions, total**	219	47.9
Definite metastatic lesions	34	7.4
Lung/LN/bone/others	14/8/8/4	3.1/1.8/1.8/0.9
Possible metastatic lesions	45	9.8
Lung/LN/bone/GI tract/others	18/9/7/4/7	3.9/2/1.5/0.9/1.5
Definite benign lesions	140	30.6
Lung/LN/bone/GI tract/others	40/30/23/21/26	8.8/6.6/5/4.6/5.7
**Final findings, total**	219	47.9
Truly metastatic lesions	45	9.8
Lung/LN/bone/others	17/12/10/6	3.7/2.6/2.2/1.3
Truly benign lesions	174	38.1
Lung/LN/bone/GI tract/others	55/35/28/23/32	12/7.7/6.1/5/7

Abbreviation: PET/CT, positron emission tomography-computed tomography; LN, lymph node; GI, gastrointestinal.

Definitive metastases were observed in 34 patients (7.4%), including 14 lung lesions, eight in LN, and eight bony metastases. Non-definitive, potentially metastatic lesions were identified in 45 (9.8%) patients (lung, 18; LN, 9; bone, 7). Follow-up imaging (within a minimum of 3 months) by chest CT, abdominal CT/MRI and whole-body bone scan or MRI of bone confirmed extrahepatic metastasis in 10 (22.2%) of these 45 patients. Both size and number of LNs had increased in two patients, two patients showed lung metastases by chest CT, and positive whole-body bone scan or bone MRI findings were noted in two patients. Of the 79 patients with definitive (n = 34) or potential (n = 45) metastases by initial ^18^F-FDG PET/CT, 44 patients had true metastatic disease.

On the other hand, of 378 patients with unequivocally normal (n = 238) or benign (n = 140) initial ^18^F-FDG PET/CT results, only one patient had a verifiable lung metastasis, without a potential or definite lesion on initial ^18^F-FDG PET/CT. Hence, 45 of the 457 patients had true metastases. In this setting, ^18^F-FDG PET/CT performance indices were as follows: sensitivity, 0.978 (44/45); specificity, 0.915 (378/413); positive predictive value (PPV), 0.556 (44/79); negative predictive value (NPV), 0.997 (378/379); and accuracy, 0.921 ([44+378]/[44+35+1+378]).

Among the 45 patients with true metastases, 28 patients had extrahepatic disease on dynamic CT of liver, including infradiaphragmatic LN metastases (n = 10), perihepatic only (n = 7) or coexistent with supradiaphragmatic (n = 2) or aortocaval (n = 1) metastases; lung metastases (n = 6); bone metastases (n = 6); and other sites (pancreas, spleen, adrenal gland, peritoneal cavity [n = 6]). In four patients, lung metastases were evident by chest x-ray. In six patients, lung metastases were evident by both liver dynamic CT and chest x-ray. Additional metastases were detected in 7 (1.5%) of the 457 patients by ^18^F-FDG PET/CT (Table 3).

**Table 3:** Extrahepatic metastases shown by liver dynamic CT or chest X-ray.

Images	Metastatic lesions on PET/CT (n = 45)
On liver dynamic CT, n (%)	28 (62.2)
On chest X-ray, n (%)	4 (8.9)
On both, n (%)	6 (13.3)
Invisible on both, n (%)	7 (15.6)

Abbreviation: CT, computed tomography; PET, positron emission tomography.

### Impact of ^18^F-FDG PET/CT on HCC stage

**Figure 1:**
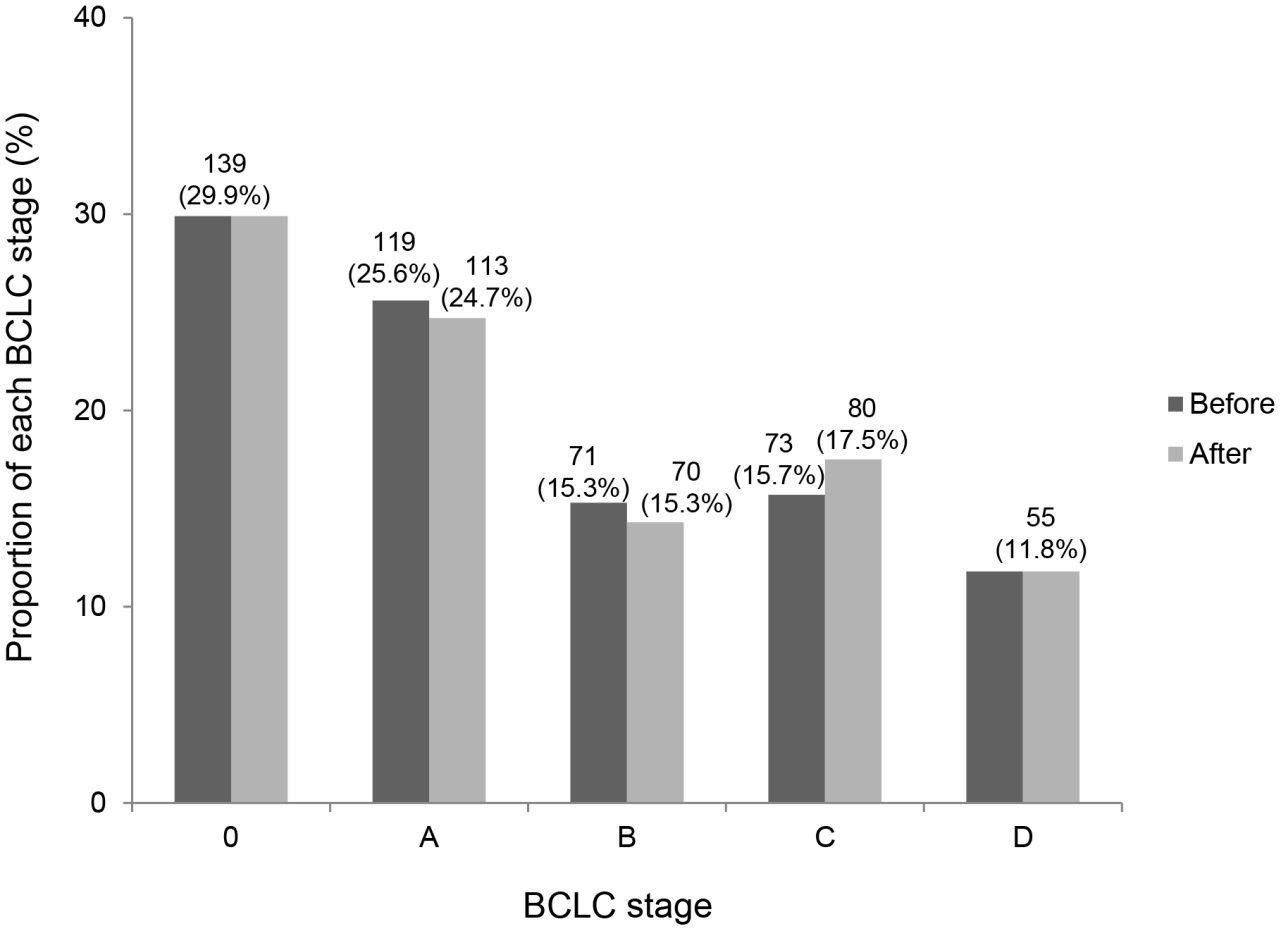
Impact of ^18^F-FDG PET/CT on BCLC stage. Seven patients showed a shift in BCLC stage from A to C (6/119, 5.0%) and B to C (1/71, 1.4%). Before, BCLC stage before ^18^F-FDG PET/CT; after, BCLC stage after ^18^F-FDG PET/CT.

BCLC staging at baseline, prior to ^18^F-FDG PET/CT, was as follows: stage 0, 139 (29.9%); stage A, 119 (25.6%); stage B, 71 (15.3%); stage C, 73 (15.7%); and stage D, 55 (11.8%). After ^18^F-FDG PET/CT, revisions were as follows: stage 0, 139 (29.9%); stage A, 113 (24.7%); stage B, 70 (15.3%); stage C, 80 (17.5%); and stage D, 55 (11.8%). Seven (1.5%) of the 457 patients showed a shift in BCLC stage from A to C (6/119, 5.0%) and B to C (1/71, 1.4%), whereas none of the patients classified as BCLC stage 0, C, or D disease by dynamic CT of liver showed a shift in BCLC stage after ^18^F-FDG PET/CT (*P*<0.001) (Fig 1).

Prior to ^18^F-FDG PET/CT, 163 patients (35.7%) failed to meet Milan criteria. That figure increased to 168 (36.8%) following ^18^F-FDG PET/CT evaluations, for a total of 5 additional patients (1.1%) who were deemed ineligible by Milan criteria.

**Figure 2:**
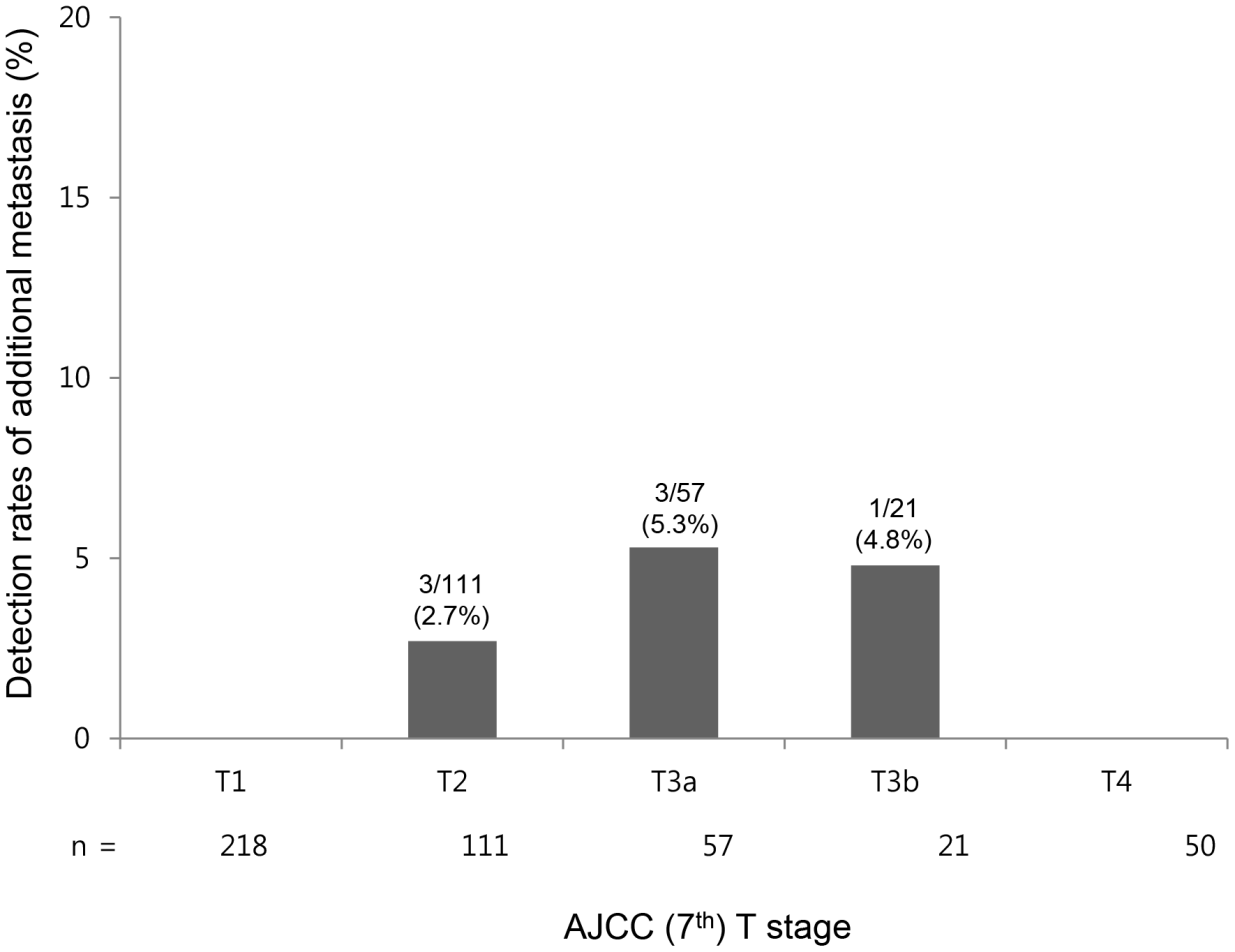
Impact of ^18^F-FDG PET/CT on AJCC (7^th^) T classification. ^18^F-FDG PET/CT detected extrahepatic HCC metastases in 2.7%, 5.3%, and 4.8% of patients with AJCC classification T2, T3a, and T3b tumors, respectively, but not in any patient with T1 or T4 classified HCC.

Baseline AJCC staging, prior to ^18^F-FDG PET/CT, was as follows: stage I, 206 (44.3%); stage II, 89 (19.1%); stage IIIA, 46 (9.9%); stage IIIB, 23 (4.9%); stage IIIC, 1 (0.2%); stage IVA, 59 (12.7%); and stage IVB, 33 (7.1%) After ^18^F-FDG PET/CT, revisions were as follows; stage I, 206 (44.3%); stage II, 89 (19.1%); stage IIIA, 42 (9.2%); stage IIIB, 20 (4.4%); stage IIIC, 1 (0.2%); stage IVA, 62 (13.6%); and stage IVB, 37 (8.1%). Additional metastases were discovered by ^18^F-FDG PET/CT in patients with T2 (3/111, 2.7%), T3a (3/57, 5.3%), and T3b (1/21, 4.8%) classifications exclusively, whereas no additional metastases were noted for the patients classified as T1 or T4 (*P* = 0.04) (Fig 2). After ^18^F-FDG PET/CT, the proportions of extrahepatic metastasis according to T classification were as follows: T1, 4/218 (1.8%); T2, 10/111 (9.0%); T3a, 12/57 (21.1%); T3b, 7/21 (33.3%); T4, 12/50 (24.0%).

### Impact of ^18^F-FDG PET/CT on HCC treatment

A total of 7 patients showed shifts in BCLC stage after performing ^18^F-FDG PET/CT. Six patients who had been classified as BCLC stage A before ^18^F-FDG PET/CT finally turned out to be classified as BCLC stage C due to LN (n = 2), bone (n = 3), and lung (n = 1) metastases after ^18^F-FDG PET/CT. Five patients among them were treated with systemic chemotherapy (ie, sorafenib or 5-fluorouracil plus mitomycin) and additional radiotherapy for bone metastasis. Another one patient with lung metastasis was successfully treated with video-assisted thoracoscopic surgery (VATS). A single 0.8 cm-sized round pulmonary nodule had been found in CT imaging, regarded as a benign inflammatory nodule. However after ^18^F-FDG PET/CT, lung metastasis was highly suspected because it showed high standardized uptake value (SUV) as high as 7.3. Fine needle biopsy was performed for the nodule. Finally, it turned out to be a true lung metastasis, histologically. After performing VATS, he could undergo living donor LT.

One patient who had been classified as BCLC stage B before ^18^F-FDG PET/CT turned out to be classified as BCLC stage C due to LN metastasis after^18^F-FDG PET/CT. Sorafenib was the treatment option for the patient.

## Discussion

In the initial staging of HCC, ^18^F-FDG PET/CT was of benefit for this cohort, impacting those patients with BCLC stage A or B disease and T2 or T3 AJCC classifications. Metastatic lesions by ^18^F-FDG PET/CT and not evident by dynamic CT of liver or chest x-ray accounted for 1.5% of patients, resulting in some revisions (BCLC, 1.5%; Milan criteria, 1.1%). ^18^F-FDG PET/CT showed a high negative predictive value (99.7%). This is the first large-scale retrospective cohort analysis to evaluate the contribution of ^18^F-FDG PET/CT in initial work-up of HCC by tumor staging conventions.

Clinical studies and autopsy findings indicate that extrahepatic metastases are not unusual in patients with HCC. Sites frequently involved are lung (18–53.8%), bone (5.8–38.5%), and LN (26.7–53%). Other potential sites of involvement are adrenal gland, peritoneum, skin, brain, and muscle [11], [20]. Locoregional therapies, such as LT, are not indicated in patients with extrahepatic metastases, the latter constituting systemic disease. Precision in staging of HCC is therefore critical for appropriate therapeutic choices, especially if LT is contemplated.

^18^F-FDG-based PET is presently used in other oncologic contexts for diagnostic staging and monitoring of chemo- or radiotherapeutic efficacy [16], [21]. However, the sensitivity of ^18^F-FDG PET in primary HCC is very low. Accumulation of FDG in malignant tumors is a function of glucose-6-phosphatase activity, which varies widely for patients with HCC [22]. In an earlier study, ^18^F-FDG PET failed to detect primary hepatic malignancy and extrahepatic metastasis in liver transplant candidates [23]. ^18^F-FDG PET may also be superfluous in patients with early-stage HCC, where the incidence and detection rates of extrahepatic metastasis are low [24]–[27]. Moreover, if extrahepatic metastases are documented by dynamic CT of liver, further delineation by ^18^F-FDG PET has no effect on choice of treatment.

However, in our study, 7 (1.5%) of the 457 patients assessed by dynamic CT of liver showed a shift in BCLC stage from A to C (6/119, 5.0%) and B to C (1/71, 1.4%), based on ^18^F-FDG PET/CT results (BCLC stage 0 and stage C tumors were unchanged). Therefore, our findings indicate that ^18^F-FDG PET/CT has value in initial staging of early (stage A) or intermediate (stage B) HCC, as determined by dynamic CT of liver and BCLC or AJCC classifications.

False-positive accumulation in inflammatory lesions is one limitation of ^18^F-FDG PET/CT use in the setting of HCC. This is well-known drawback, so caution is needed to avoid erroneous interpretation [28]. Among the 174 truly benign lesions encountered in our study, 34 were initially assessed as definitive (n = 2) or potential (n = 32) metastases by ^18^F-FDG PET/CT. Nonetheless, recent studies have shown that ^18^F-FDG PET was useful for tumor characterization and prognostication [29]–[31]. The maximum SUV correlated inversely with disease-free survival time and recurrence risk [30]. Likewise, tumor progression and aggressiveness of HCC may be extrapolated from PET studies [32], [33]. According to reports, poorly differentiated HCC shows greater uptake of ^18^F-FDG, and a positive correlation between uptake and rapid tumor growth has been demonstrated [34].

Also, clinical studies have reported that PET/CT was useful in predicting postoperative tumor recurrence and prognosis of the patients with HCC undergoing hepatic resection or LT [35]–[37]. Therefore, PET/CT might be justified not only for staging shown by this study but also for tumor characterization and prognostication. Especially, initial HCC staging work-ups including PET/CT for extrahepatic lesions should be considered for instances of planned hepatic resection or LT. Add to this our finding that the patients with BCLC stage A and B disease actually benefitted from ^18^F-FDG PET/CT, through discovery of otherwise undetected metastases, then it is only when these patient subsets are destined for surgery or transplantation that the cost of ^18^F-FDG PET/CT may be justified.

Our study has several limitations. Among the consecutive 457 patients in this study who underwent ^18^F-FDG PET/CT at the time of HCC diagnosis, many of them had been with early stages (BCLC stage 0 or A) who were considered to be treated with curative modalities including surgical resection and locoregional therapies. Therefore, the number of the patients with advanced stage (BCLC stage C) or end stage (BCLC stage D) included in this study was relatively smaller than that of the patients with early stages, which is a limitation of retrospective cohort study. Moreover, 72% of the patients included in this study had been under regular surveillance for HCC, which might lead to the earlier stages of study subjects. Larger multi-center prospective studies are warranted to confirm these promising results.

In conclusion, our findings indicate that routine staging of HCC (ie, dynamic CT of liver and chest x-ray) gains information from added ^18^F-FDG PET/CT in patients with BCLC stage A and stage B disease or with AJCC T2 and T3 classifications with a high-negative-predictive value. HCCs at very early stages have low prevalence of extrahepatic metastases, which means that ^18^F-FDG PET/CT may be unessential for initial staging of patients with very early HCC, in the absence of clinical suspicion for distant metastases with any symptoms or signs. However, ^18^F-FDG PET/CT has value in initial staging of early (BCLC A) or intermediate HCC (BCLC B), especially if hepatic resection or LT is planned.
